# Carotid artery stenting with open vs closed stent cell configurations in the CREST-2 Registry

**DOI:** 10.1016/j.jvs.2025.02.025

**Published:** 2025-02-28

**Authors:** Brajesh K. Lal, Gary S. Roubin, James F. Meschia, Michael Jones, Donald V. Heck, W. Charles Sternbergh, Herbert D. Aronow, Carlos Mena-Hurtado, George Howard, Minerva Mayorga-Carlin, John D. Sorkin, Thomas G. Brott

**Affiliations:** a Department of Vascular Surgery, University of Maryland School of Medicine, Baltimore; b CREST-2 Interventional Management Committee, Jackson; c Department of Neurology, Mayo Clinic, Jacksonville; d Department of Cardiology, Baptist Health, Lexington; e Department of Radiology, Novant Health, Winston-Salem; f Department of Vascular Surgery, Ochsner Health, New Orleans; g Division of Cardiovascular Medicine, Henry Ford Hospital, Detroit; h Department of Medicine, Michigan State University College of Human Medicine, East Lansing; i Vascular Outcomes Program (VAMOS) & Section of Cardiology, Department of Internal Medicine, Yale University, New Haven; j Department of Biostatistics, University of Alabama at Birmingham, Birmingham; k Department of Medicine, University of Maryland School of Medicine, Baltimore; l Baltimore VA Geriatrics Research Education and Clinical Center, Baltimore.

**Keywords:** Carotid stenting, Registry, Stenosis, Stent cell configuration, Stroke

## Abstract

**Objective:**

Intraprocedural atheroembolization during carotid artery stenting (CAS) can be reduced through careful patient selection, consideration of vascular anatomy and lesion characteristics, operator and institutional experience, peri-procedural antithrombotic and antiplatelet therapy, and use of embolic protection. However, CAS can also result in stroke as the stent is deployed and embolic protection withdrawn. The free-cell area of most closed-cell stents is <5 mm^2^, and ≥5 mm^2^ for open-cell stents. The larger area may permit escape of more atheromatous debris. Comparisons of clinical outcomes between closed-cell and open-cell stents have been inconclusive. The aim of this study is to compare clinical outcomes associated with CAS using open-cell vs closed-cell stents.

**Methods:**

The CREST-2-Registry (C2R) enrolls asymptomatic and symptomatic patients for whom CAS is favored because of high risk for surgery or patient preference. C2R implements operator- and site-credentialing, careful lesion selection, and standardized procedural protocols. Patient characteristics, procedural details, and outcomes are recorded. Interventionists may use United States Food and Drug Administration-approved devices including open-cell stents (Rx Acculink [Abbott Vascular], Precise Pro Rx [Cordis-Cardinal Health], and Protégé Rx [Medtronic/Covidien]), or closed-cell stents (XACT [Abbott Vascular] and Wallstent Monorail Endoprosthesis [Boston Scientific]). Multivariable logistic regression was used to assess relate stent cell configuration to peri-procedural (30-day) stroke or death (SD).

**Results:**

Of 5307 procedures performed by 163 interventionists across 101 clinical centers, 2054 (38.7%) received open-cell stents, and 3253 (61.3%) received closed-cell stents. In the periprocedural period, 91 patients (1.7%) experienced a stroke (3 were fatal), and 16 patients died without experiencing strokes (0.4%). After adjusting for age, sex, symptomatic status, and case urgency, and for effect-modification by indication, periprocedural SD was significantly higher when an open-cell stent was placed in a primary lesion compared with closed-cell stents (3.5 events per 100 procedures using open-cell stents [95% confidence interval [CI], 2.6–4.7] vs 2.2% [95% CI, 1.6–3.0] using closed-cell stents (odds ratio, 1.59; 95% CI, 1.13–2.23; *P* < .01). Periprocedural SD was not significantly different between stent types when placed in a restenotic lesion (1.2% [95% CI, 0.4–3.3]) using open-cell stents vs 4.0% (95% CI, 2.2–7.2) using closed-cell stents (odds ratio, 0.31; 95% CI, 0.09–1.01; *P* = .052).

**Conclusions:**

Stent design influences periprocedural stroke or death in carotid stenting. Closed-cell stents are associated with a lower event rate when treating primary atherosclerosis, but not in the setting of restenosis.

Intraprocedural atheroembolization during carotid artery stenting (CAS) can be reduced through careful patient selection, consideration of vascular anatomy and lesion characteristics, operator and institutional experience, periprocedural antithrombotic and antiplatelet therapy, and the use of embolic protection.^1^ However, CAS can also result in post-procedural atheroembolic stroke after the stent is deployed and embolic protection is withdrawn. Of the 48 strokes occurring within the 30-day periprocedural period in patients undergoing CAS in CREST, 29 occurred on the procedure day, 10 occurred on days 1 to 7, and nine occurred on days 8 to 30.^2^ Stent deployment compresses the plaque and traps the crushed atheroma behind its scaffolding.^3^ As stents expand, plaque protrusion can occur through their interstices, and lead to late atheroembolization.^4,5^ The risk of embolization of the protruded atheroma long after stent deployment may contribute to the continued risk of stroke reported several days after CAS.

The free cell area of most closed-cell stents is <5 mm^2^, whereas that of open-cell stents is ≥5 mm^2^. Open-cell stents (with the larger free cell area) may permit escape of atheromatous debris more often than stents with a closed-cell design.^6–8^ Comparisons of clinical outcomes between closed-cell and open-cell stents have been inconclusive.^9–15^ Most studies have failed to find a difference in periprocedural stroke or death (SD) rates between the two stent cell types, whereas some have found higher SD rates with open-cell and others with closed-cell stents. Periprocedural event rates after CAS are low, and available studies are challenged by small sample sizes, non-standardized data-collection, and confounding from varied operator experience and patient populations.

CREST-2 is a set of two trials evaluating whether the addition of carotid revascularization (surgery or stenting) to intensive medical management affords reduced stroke or death rates among asymptomatic patients with ≥70% carotid stenosis. The accompanying CREST-2 Registry (C2R) was initiated to maintain, enhance, and ensure expertise in CAS and to facilitate rigorous credentialing of stent operators and enrollment sites.^16^ Participation in C2R is predicated on the use of standardized CAS protocols, monitoring of patient selection and outcomes, and commitment to reporting of consecutive CAS procedures. Open-cell and closed-cell stents approved for use by the United States Food and Drug Administration are available for use per interventionist preference.

The aim of this study is to compare clinical outcomes associated with CAS performed using open-cell and closed-cell stent designs in the C2R.

## METHODS

### Study design.

The C2R was approved for enrollment in September 2014. It enrolls patients with carotid stenosis warranting CAS. The protocol was reviewed and approved by the University of Maryland School of Medicine Institutional Review Board and granted waiver of written consent. The protocol is registered with clinicaltrials.gov (NCT02240862). Details of patient eligibility criteria, site selection, and physician credentialing have been described previously.^16^ Briefly, interventionists are reviewed and credentialed by a multispecialty Interventional Management Committee, and sites are reviewed and approved by a multispecialty Site Selection Committee. Patients can be asymptomatic or symptomatic (having experienced a stroke, transient ischemic attack [TIA], or amaurosis fugax within 180 days before the procedure), and can be standard surgical risk, high anatomic risk, or high physiologic risk for carotid endarterectomy. Exclusions include patients <18 or >80 years of age, symptomatic patients with a stenosis <50%, asymptomatic patients with a stenosis <70%, modified Rankin score >3, severe (New York Heart Association class IV) congestive heart failure, chronic obstructive pulmonary disease on home oxygen, severe (Child Pugh class D) liver failure, cancer with metastatic spread or undergoing chemotherapy, or dementia greater than mild. This analysis was restricted to procedures performed for either primary atherosclerosis or restenosis (after a prior endarterectomy or stent procedure) and excluded stenoses from less common etiologies such as trauma, dissection, and fibromuscular dysplasia. The analytic cohort was further restricted to procedures that used one single-layer open-cell or closed-cell stent, excluding balloon angioplasties where no stent was placed, procedures that utilized multiple stents, and procedures that utilized a dual-layer stent (eg, covered or micro-mesh). Among patients who underwent multiple procedures during the study period, only the first eligible procedure was included in the analysis.

### Procedural protocol.

After undergoing web-based training for a standardized CAS protocol, interventionists can begin enrolling in C2R.^17,18^ The training program is based on lessons learned from CREST and describes optimal patient and lesion selection, procedural technique, and reporting methodology.^1^ Physicians are strongly encouraged to use periprocedural dual antiplatelet therapy, preprocedural statins, and procedural anticoagulation; and to avoid adverse aortic arch anatomy (Type 3 or bovine arch), tortuous common or internal carotid arterial anatomy (two or more 90° turns), marked concentric calcification, and noncontiguous or long (>3 cm) lesions. Prolonged filter dwell times, overuse of intra-arterial contrast runs, and aggressive post-stenting angioplasty with large balloons (diameter >5.0 mm) are discouraged. The choice of anesthesia, access site, and revascularization devices are left to the discretion of the interventionist, though stents and embolic protection devices must be United States Food and Drug Administration-approved, and the use of some form of embolic protection is mandatory.

Elective procedures were those performed on an outpatient basis or during a subsequent hospitalization predicated on the convenience of the interventionist and patient. Urgent procedures were those performed within 2 days of diagnosis. A patient requiring an urgent procedure warranted immediate admission; if already admitted, the procedure was performed prior to discharge. Emergency procedures were usually performed within 6 hours of diagnosis. The distinction between urgent and emergency procedures can vary across institutions; we therefore grouped urgent and emergency procedures together in this analysis.

### Data collection.

The C2R data collection, management, and quality assurance protocols have been described previously.^16^ Briefly, information on patient demographics, vascular risk factors, lesion characteristics, procedural details, and peri-procedural outcomes are collected. Race and ethnicity are self-reported. Enrollment into C2R is ongoing. The C2R Administrative Center performs data quality checks to ensure accuracy and completeness of all information collected. Data were obtained from the C2R database describing CAS procedures performed between September 17, 2014, and February 28, 2023. Data were analyzed by an independent statistical team with no input from funding agencies. Industry partners had no access to raw C2R data.

### Outcome measures.

The periprocedural period begins on the day of the procedure and ends 30 days later. Adverse events occurring during this period are recorded. The primary outcome was a composite of periprocedural stroke or death (SD). Composite outcomes count each patient who experienced one or more of the component events as a single event. A stroke was defined as a clinically detected sudden neurological deficit lasting >24 hours consistent with stroke, or an increase of ≥2 points on the National Institutes of Health Stroke Scale.^19,20^ Neurologic examinations and National Institutes of Health Stroke Scale assessments were performed at baseline and at follow-up. All deaths were included regardless of cause.

### Statistical analysis.

Patients in the analytic cohort were categorized into two groups based on the configuration of the stent they received: open-cell (Rx Acculink [Abbott Vascular], Precise Pro Rx [Cordis-Cardinal Health], and Protégé Rx [Medtronic/Covidien]), and closed-cell (XACT [Abbott Vascular] and Wallstent Monorail Endoprosthesis [Boston Scientific]).

To compare baseline characteristics in the two groups, the Pearson χ^2^ test was used for categorical variables, and the Kruskal-Wallis test was used for continuous variables. Unbalanced characteristics were considered potential confounders if they had a significant association with the primary outcome; confounders were included as main effects in the regression model as described below. Interactions between each baseline characteristic and the effect of the stent cell configuration on the primary outcome were investigated using the Breslow-Day test for homogeneity of the odds ratios (ORs). Characteristics that led to heterogenous ORs were considered effect modifiers; effect modifiers were included as both main effects and interaction terms in the regression model as described below.

To assess the effect of stent cell configuration on peri-procedural stroke or death, we used multivariable logistic regression weighted by the inverse probability of receiving an open-cell stent. Generalized estimating equations of Liang and Zeiger were used with an exchangeable correlation structure to account for serial autocorrelation of procedures performed by the same physician and/or in the same hospital; the unit of repeated measures was physician × institution.^21^ The regression model had six main effects and one interaction term; the main effects were stent cell configuration (open vs closed), age, sex (male vs female), symptomatic status (symptomatic vs asymptomatic), case urgency (elective vs urgent or emergency), and indication (primary atherosclerosis vs restenosis); the interaction term was stent cell configuration × indication. Although there was no evidence of effect modification by symptomatic status, there was evidence of potential confounding. Symptomatic patients were more likely to get closed-cell stents (*P* < .01) and also more likely to experience an SD (*P* < .01). We used the Pearson χ^2^ test to assess those relationships. Because symptomatic status had a significant association with the exposure (stent cell type) as well as the outcome (SD), it was added as a main effect in the multivariable regression model. The weights (inverse probability of getting an open-cell stent) were calculated through a separate logistic model with stent cell configuration as the outcome and the following five main effects: age, sex, symptomatic status, and case urgency. Results are presented as ORs and 95% confidence intervals (CIs) stratified by the effect modifier (ie, primary lesions separate from restenotic lesions).

Sensitivity analyses were performed to rule out residual confounding by baseline characteristics and procedural details that did not meet the criteria described above to be considered confounders or effect modifiers. These models assessed the impact of adjusting for additional main effects in the multivariable regression model. A two-tailed *P* value ≤ .05 was considered statistically significant. Data analyses were performed using SAS version 9.4 (SAS Institute).

## RESULTS

### Demographic and clinical characteristics of the analytic cohort.

**Fig 1.**
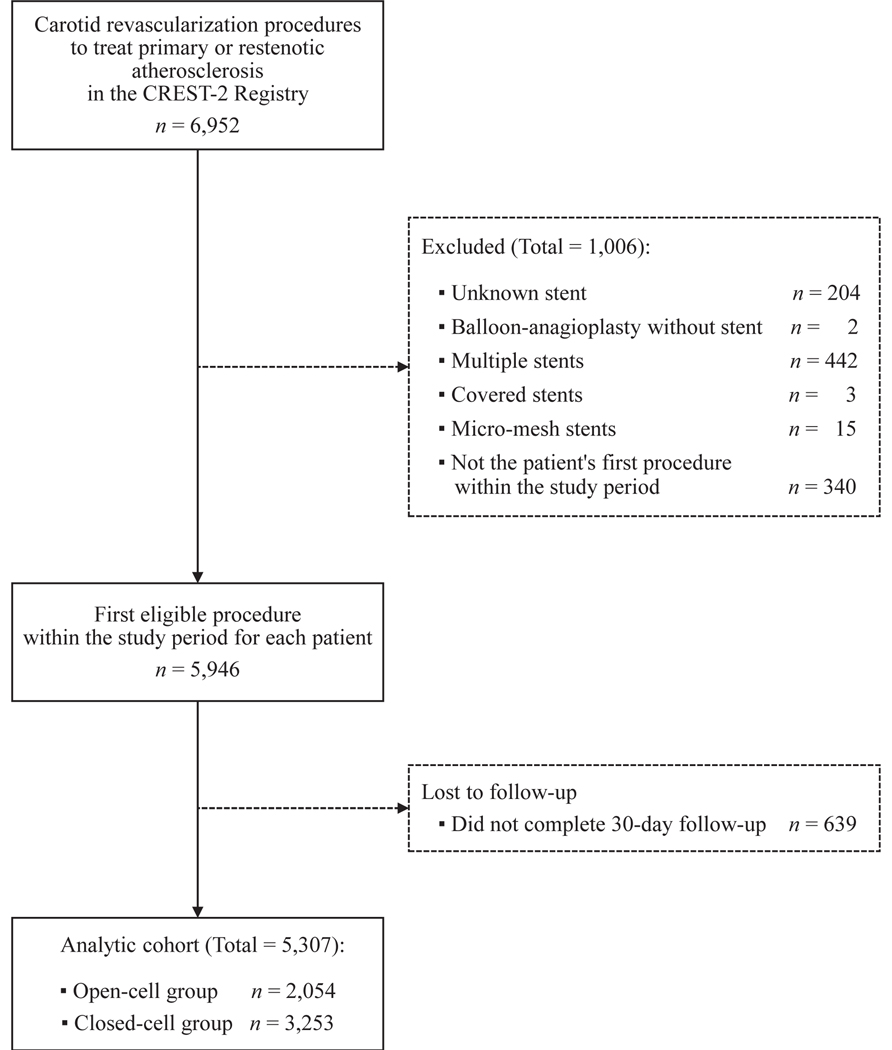
Flow diagram of analytic cohort. The analytic cohort is a subset of data extracted from the CREST-2 Registry (*C2R*), which was initiated to maintain, enhance, and ensure expertise in carotid artery stenting (CAS) and to facilitate rigorous credentialing of stent operators and enrollment sites for the CREST-2 trial. In addition to the C2R eligibility criteria, this analysis was restricted to procedures performed for either primary atherosclerosis or restenosis (excluding less common etiologies like trauma, dissection, and fibromuscular dysplasia) that used one single-layer open-cell or closed-cell stent. Among patients who underwent multiple procedures during the study period, only the first eligible procedure was included.

Of 6952 CAS procedures entered in C2R, we excluded 204 for unknown stents, three for covered stents, 15 for micro-mesh stents, two for balloon angioplasties, 442 for more than one stent in same procedure, 340 for second procedure on same patient, and 639 for no follow-up visit. The remaining 5307 patients were divided into two groups: 2054 patients (38.7%) received an open-cell stent, and 3253 (61.3%) received a closed-cell stent (Fig 1). A total of 4289 patients (80.8%) were treated for primary atherosclerosis, whereas 1018 (19.2%) were treated for restenosis. We compared patients that did not follow-up (n = 639) with our analytic cohort with follow-up (n = 5307) and found no significant differences in their clinical or demographic characteristics.

**Fig 2.**
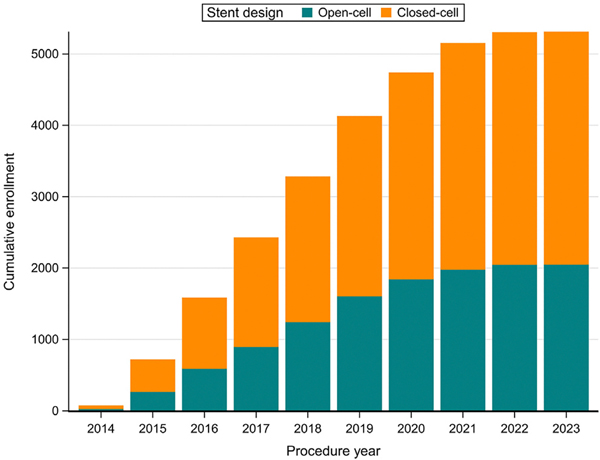
Enrollment in the CREST-2 Registry (C2R). Cumulative enrollment of carotid artery stenting (CAS) procedures to treat primary or restenotic atherosclerosis using a single-layer open-cell or closed-cell stent. The total of 5307 procedures were performed by 163 interventionists. Each month, 1 to 40 procedures using an open-cell stent (median, 21) plus 1 to 65 procedures using a closed-cell stent (median, 35) were enrolled.

**Fig 3.**
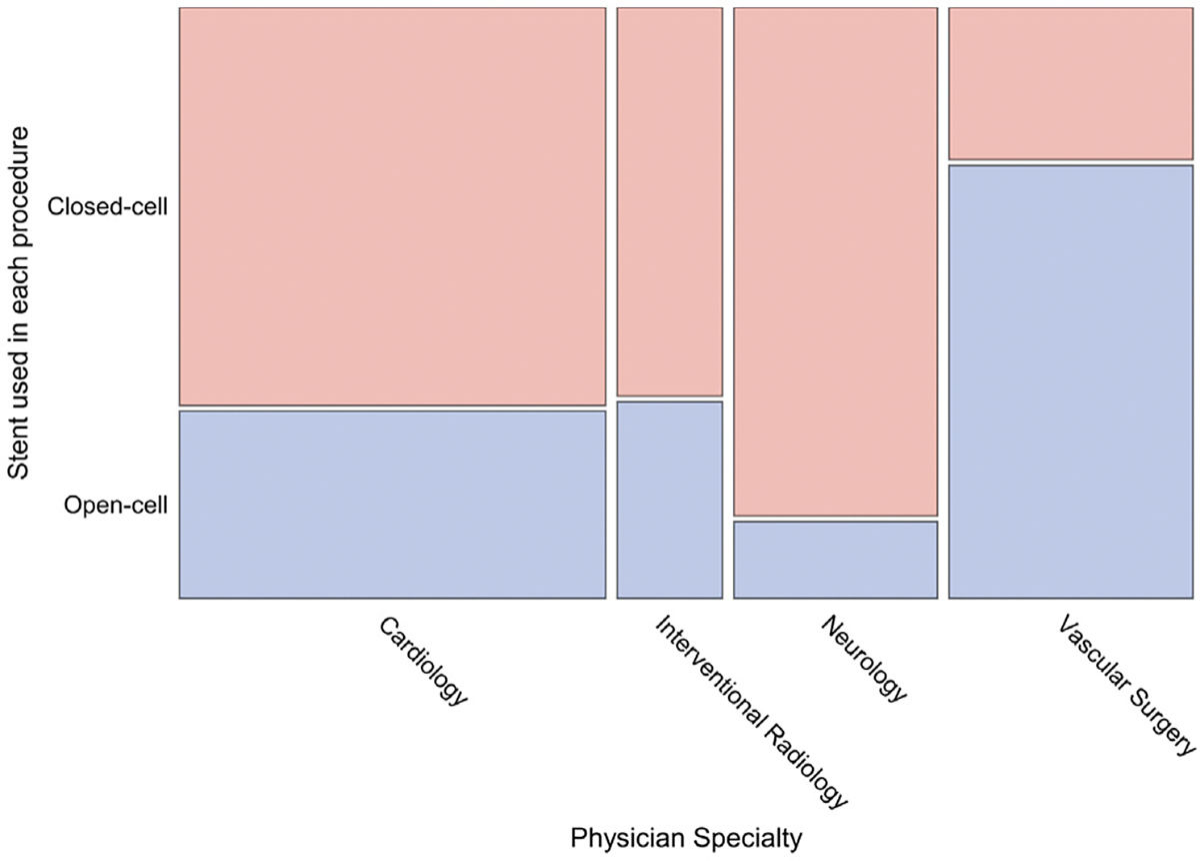
Proportion of procedures done by physician specialty. Open-cell stents were more common among procedures by vascular surgeons (74%). Closed-cell stents constituted 68% of procedures by cardiologists, 87% by neurologists, and 66% by interventional radiologists.

Fig 2 shows the cumulative enrollment in C2R over time. The registry enrolled a median of 21 open-cell cases per month (interquartile range [IQR], 11–28; range, 1–40) and 35 closed-cell cases per month (IQR, 19–44; range, 1–65). Procedures were performed by 163 interventionists: 70 cardiologists (2311 procedures), 39 vascular surgeons (1322 procedures), 35 neurologists and neurosurgeons (1103 procedures), and 19 radiologists and neuroradiologists (571 procedures). Open-cell stents were more common among procedures by vascular surgeons (74%). Closed-cell stents constituted 68% of procedures by cardiologists, 87% by neurologists, and 66% by interventional radiologists. This trend was driven partially by transcarotid artery revascularization procedures, which were performed primarily with open-cell stents (96.7%) and by vascular surgeons (94.1%). However, a majority of physicians from each specialty contributed procedures to both groups; 55.7% of cardiologists, 47.4% of interventional cardiologists, 62.9% of neurologists, and 64.1% of vascular surgeons performed at least one procedure with an open-cell stent and at least one procedure with a closed-cell stent (Fig 3).

There were differences in baseline characteristics between the open- and closed-cell groups, although most were numerically small (Table I). The open-cell group was older, had more non-White and Hispanic patients, a higher prevalence of hypertension and coronary artery disease, and more restenosis as the indication for the procedure, vs the closed-cell group. The open-cell group had fewer diabetics, symptomatic patients, and nonelective procedures. Patients in the open-cell group were also more likely to get preoperative antiplatelets and general anesthesia, but less likely to get postoperative dual antiplatelet therapy (DAPT) compared with patients in the closed-cell group. The most common access site was the femoral artery in both groups, but most transcarotid procedures used open-cell stents, and most procedures that used other access vessels (brachial and radial) used closed-cell stents. Similarly, the most common type of embolic protection was distal filter in both groups, but transcarotid flow reversal was used more often with open-cell stents, and transfemoral flow reversal was used more frequently with closed-cell stents.

Only symptomatic status and case urgency met our criteria for confounding, having a significant association with both the stent cell configuration and periprocedural SD (*P* < .01). Age and sex are recognized as important risk factors in carotid artery revascularization and were therefore also considered potential confounders.^1^ Only indication met our criteria for effect modification; the effect of stent cell configuration on periprocedural SD was found to be significantly different for primary lesions compared with restenotic lesions (*P* = .01). There were no other significant differences or interactions by baseline characteristics (Supplementary Fig, online only). Although there was a statistical difference in DAPT use between groups, the numeric difference was small and resulted in no statistical evidence of confounding.

### Primary outcome: periprocedural stroke or death.

In our cohort, 91 patients experienced a stroke, three of which were fatal, and 16 additional patients died without experiencing strokes. In unadjusted analyses (Table II), for the entire cohort, the composite SD rate was 2.0%, and there was no difference in SD rates between the open-cell and closed-cell groups. Among patients treated for primary atherosclerosis, SD occurred in 38 of 1568 patients receiving open-cell stents and in 51 of 2721 patients receiving closed-cell stents. Among patients treated for restenosis, SD occurred in four of 486 patients receiving open-cell stents and 14 of 532 patients receiving closed-cell stents.

**Fig 4.**
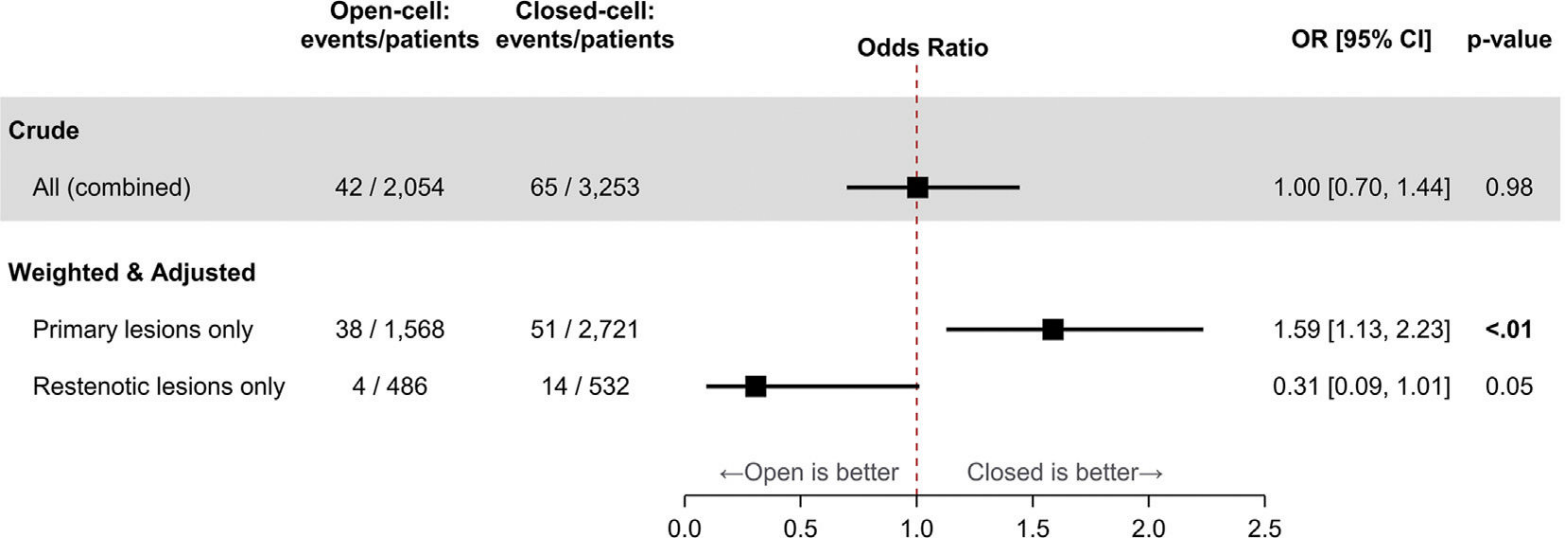
Odds of periprocedural stroke or death, open-cell vs closed-cell stents. Among patients treated for primary atherosclerosis, the adjusted stroke or death (SD) rate was higher in patients receiving open-cell stents compared with closed-cell stents. Among patients treated for restenosis, event rates were not significantly different between stent groups. Odds ratios (*ORs*), 95% confidence intervals (*CIs*), and *P*-values were estimated through logistic regression models using generalized estimating equations to account for serial autocorrelation of procedures performed by the same physician in the same hospital. *Crude,* Unweighted and unadjusted model to compare outcomes by stent cell configuration. *Weighted & adjusted*, inverse probability of treatment weighting (IPTW); adjusted for age, sex, symptomatic status, case urgency, and the interaction between stent cell configuration and indication. Events refers to the number of patients who experienced stroke or death; each patient is counted only once even if the patient experienced more than one event.

After accounting for confounding by age, sex, symptomatic status, and case urgency, and for effect modification by indication (Fig 4), the SD rate among patients treated for primary atherosclerosis was significantly higher among those receiving an open-cell stent (3.5%; 95% CI, 2.6%–4.7%) compared with a closed-cell stent (2.2%; 95% CI, 1.6%–3.0%; OR, 1.59; 95% CI, 1.13–2.23; *P* < .01). The SD rates in patients treated for restenosis were not significantly different between those receiving an open-cell vs a closed-cell stent (rates 1.2%; 95% CI, 0.4%–3.3% vs 4.0%; 95% CI, 2.2%–7.2%; OR, 0.31; 95% CI, 0.09–1.01; *P* = .052).

### Sensitivity analyses.

Adjusting the analysis for embolic protection type, preoperative DAPT, postoperative DAPT, or physician specialty by adding them as main effects in the multivariable logistic regression model did *not* change the results in any important manner. We also assessed early-vs later-year experience in CAS and found no change in trends for SD rates across the 10-year experience in C2R (data not shown).

## DISCUSSION

Our results from an analysis of 5307 procedures suggest that stent design influences periprocedural event rates in CAS. The overall periprocedural SD rate in symptomatic or asymptomatic patients undergoing CAS in the C2R was low. Among patients treated for primary atherosclerosis, the adjusted SD rate was higher in patients receiving open-cell stents compared with closed-cell stents (3.5%; 95% CI, 2.6%–4.7% vs 2.2%; 95% CI, 1.6%–3.0%). Event rates were not significantly different between stent groups in patients treated for restenosis.

The C2R cohort is well-positioned to assess the relationship between stent design and SD rates from CAS because other potential factors leading to peri-procedural atheroembolization were well-accounted for. Atheroembolization primarily occurs during three different time periods: during traversal of hardware to reach the lesion, during treatment of the lesion, and during the early post-procedural period. The steps involved in reaching the lesion can cause embolization from non-carotid plaque while navigating a diseased aorta. These steps precede introduction of the stent and are therefore agnostic to the type of stent eventually used. The steps are, however, influenced by patient selection and operator technique. In C2R, patient and lesion selection were carefully monitored and standardized, and interventionists were rigorously evaluated to maximize expertise, thereby reducing potential confounding by these factors. The steps involved in treatment of the lesion such as lesion wiring, placement of embolic protection, balloon inflation, positioning and releasing the stent, and balloon-assisted stent expansion were performed in a standardized fashion, thereby reducing chances for large differences in atheroembolization during this step. In the post-procedural period, plaque protrusion from continued stent expansion for several days after deployment can lead to atheroembolization. This is potentially influenced by stent cell configuration. Our results are therefore most reflective of atheroembolic stroke risk in the post-deployment stage of CAS. Our results suggest that the containment of atherosclerotic plaque material is more effectively achieved by closed-cell compared with open-cell stent designs.

Flexibility and scaffolding are two important yet competing stent characteristics, and are driven, in part, by the size of stent interstices (free cell area). A balance is desired between the former, which dictates how well the stent travels and conforms to the lesion, and the latter, which dictates how well the stent supports the lumen and retains atherosclerotic debris. The free cell area of most closed-cell stents are <5 mm^2^ (eg, Wallstent [1.08 mm^2^] and Xact [2.74 mm^2^]), whereas that of open-cell stents are >5 mm^2^ (eg, Precise [5.89 mm^2^], Acculink [11.48 mm^2^], and Protégé [10.71 mm^2^]).^6^ In vitro studies report that the larger cell sizes in open-cell stents are more likely to permit plaque protrusion and escape of debris.^8^

There is no current consensus on whether stent cell design impacts the likelihood of periprocedural SD, thereby preventing evidence-based clinical decision-making. A single-institution experience reported higher composite SD or TIAs in open- vs closed-cell stents.^6^ Pooled experience from 10 European centers found no difference in composite SD between open- vs closed-cell stents (3.1%; 95% CI, 2.3%–3.9% vs 2.4%; 95% CI, 1.7%–3.1%; *P* = .38).^9^ Similarly, a pooled United States experience (National Cardiovascular Data Registry) reported no difference in SD by stent type after adjusting for intergroup differences.^10^ Conversely, another pooled experience from United States centers (Vascular Quality Initiative) reported that in-hospital composite SD or TIA rates were lower for open- vs closed-cell stents (2.46% vs 3.16%; OR, 0.674; 95% CI, 0.460–0.987) after adjusting for intergroup differences.^11^ The only two randomized comparisons of open-cell vs closed-cell stents did not detect differences in SD but were underpowered.^12,13^ More recently, two meta-analyses of 34^14^ and 66^15^ studies comparing open- vs closed-cell stents found no difference in SD between the two groups.

The conflicting findings in the literature may be due to sample size limitations or variability in interventionist experience.^22^ C2R uses rigorous selection criteria that reduces confounding by operator and site experience.^16^ Previous analyses pooled all CAS procedures regardless of indication (primary atherosclerosis vs restenosis). We found that the differences in periprocedural adverse events by stent design were most pronounced in patients treated for primary atherosclerosis. Due to the small number of restenosis cases enrolled in C2R, a type II error for the comparison in this group cannot be ruled out. Previous studies also did not account for case urgency (emergency vs non-emergency). We found that case urgency interacted significantly with the outcome and must be accounted for in any intergroup comparison of CAS.

### Study limitations.

The participation of 163 interventionists from over 100 clinical centers in C2R improves the generalizability of results. However, our study did not randomly assign stents for prespecified indications. Despite use of inverse probability weighting to assess standardized differences, our efforts to adjust for intergroup differences and confounding by indication may not have been entirely successful. A risk for unknown differences remaining unaccounted for remains. The prevalent classification of open- and closed-cell stent configurations that dichotomizes them at a threshold cell size of 5 mm^2^ is arbitrary, and assigned stents within each group do have different cell sizes. Our study was not powered to identify differences in outcomes by individual stents. Although the reversal in the direction of SD rates between open- vs closed-cell stents for restenosis lesions compared with atherosclerosis lesions is thought-provoking and biologically reasonable, the results were not statistically significant and require an analysis with a larger sample size. It is possible that open-cell stents perform better for unique patients that were not represented or characterized in the analysis. One study has suggested that closed-cell stents are more prone to malapposition and kinks, suggesting open-cell stents may be preferable for patients with highly tortuous anatomy. Larger studies with better characterization of lesion features, however, will be required to test this hypothesis.

## CONCLUSIONS

Stent design influences periprocedural event rates in carotid stenting. Our results are compelling enough to guide judicious stent selection based on stent cell configuration. The C2R implements operator and site credentialing, careful lesion selection, standardized procedural protocols, standardized periprocedural pharmacologic management, and careful data collection. These features, along with the large sample size enrolled by over 160 interventionists from over 100 clinical centers, permit a reliable comparison of outcomes in CAS performed with open- vs closed-cell stents. Closed-cell stents are associated with a lower SD rate when treating primary atherosclerosis, whereas stent design is not related to clinical outcome in the setting of restenosis.

## Supplementary Material

1

## Figures and Tables

**Table I.** Demographic and clinical characteristics

	Stent cell configuration		
	Open-cell n = 2054	Closed-cell n = 3253	*P*

Age, years	70 (65–75)	69 (63–74)	**<.01**
Lesion stenosis, %	85 (80–90)	85 (80–90)	.14
Demographics			
Sex (% female)	715 (34.8)	1135 (34.9)	.95
Race (% White)	1946 (94.7)	3126 (96.1)	**.02**
Ethnicity (% Hispanic)	89 (4.3)	88 (2.7)	**<.01**
Clinical risk factors			
Hypertension	1831 (89.1)	2829 (87.0)	**.02**
Diabetes	790 (38.5)	1352 (41.6)	**.02**
Prior or current smoker	1570 (76.4)	2479 (76.2)	.85
Coronary artery disease	1078 (52.5)	1488 (45.7)	**<.01**
Congestive heart failure	85 (4.1)	107 (3.3)	.11
Dysrhythmia	24 (1.2)	44 (1.4)	.56
Procedural details			
Lesion side (% left)	1027 (50.0)	1609 (49.5)	.70
Symptomatic status (% symptomatic)	677 (33.0)	1397 (42.9)	**<.01**
Preoperative antiplatelets	1947 (94.8)	3027 (93.1)	**.01**
Preoperative DAPT	1652 (80.4)	2551 (78.4)	.08
Postoperative DAPT	1862 (90.8)	3014 (92.8)	**<.01**
Anesthesia type			
General	534 (26.5)	123 (3.9)	**<.01**
Local or regional	1480 (73.5)	3012 (96.1)	
Indication			
Primary atherosclerosis	1568 (76.3)	2721 (83.6)	**<.01**
Ipsilateral restenosis	486 (23.7)	532 (16.4)	
Case urgency			
Elective	1904 (92.7)	2711 (83.3)	**<.01**
Nonelective (urgent or emergency)	150 (7.3)	542 (16.7)	
Access vessel			
Femoral artery	1353 (65.9)	3018 (92.8)	
Carotid artery	641 (31.2)	35 (1.1)	**<.01**
Other access vessel	60 (2.9)	200 (6.1)	
Embolic protection type			
Distal filter	1305 (63.5)	2981 (91.6)	
Transfemoral flow reversal	124 (6.0)	251 (7.7)	**<.01**
Transcarotid flow reversal	625 (30.4)	21 (0.6)	

*DAPT*, Dual antiplatelet therapy.

Data are presented as number (%) or median (interquartile range).

Boldface *P* values indicate statistical significance.

Missing data: lesion stenosis, 87; lesion side, 2; postoperative DAPT, 8; anesthesia type, 158.

**Table II.** Number of events and unadjusted event rates stratified by indication

	Entire cohort		Primary atherosclerosis		Restenotic lesions	
	Open-cell (n = 2054)	Closed-cell (n = 3253)	Open-cell (n = 1568)	Closed-cell (n = 2721)	Open-cell (n = 486)	Closed-cell (n = 532)

Stroke	35 (1.7)	56 (1.7)	31 (2.0)	43 (1.6)	4 (0.8)	13 (2.4)
Death	7 (0.3)	12 (0.4)	7 (0.4)	11 (0.4)	0 (0.0)	1 (0.2)
SD^a^	42 (2.0)	65 (2.0)	38 (2.4)	51 (1.9)	4 (0.8)	14 (2.6)

*SD*, Stroke or death.

Data is presented as number of events (rate per 100 patients).

a Composite event rates count each patient only once, even if the patient experienced more than one event.

## ARTICLE HIGHLIGHTS

- **Type of Research:** Multicenter retrospective review of prospectively collected CREST-2 Registry data
- **Key Findings:** Among 5307 patients undergoing carotid artery stenting, the use of an open-cell stent increased the risk of stroke or death when treating primary lesions (odds ratio, 1.59; 95% confidence interval, 1.13–2.23; *P* < .01) but not when treating restenotic lesions (odds ratio, 0.31; 95% confidence interval, 0.09–1.01; *P* = .05).
- **Take Home Message:** Compared with closed-cell stents, open-cell stents increased the risk of stroke or death among patients treated for primary atherosclerosis. However, when treating restenosis, the influence of stent design on clinical outcomes was not statistically significant. The authors recommend that closed-cell stents are used preferentially for primary lesions, whereas closed or open-cell stents may be considered for restenotic lesions.
