# Supplementary material for: Carotid artery stenting with open vs closed stent cell configurations in the CREST-2 Registry
Source: J Vasc Surg. Author manuscript; Available in PMC 2026 Jun 5. (PMC13238965; doi:10.1016/j.jvs.2025.02.025)
Supplement: 1 [file NIHMS2172117-supplement-1.pdf]

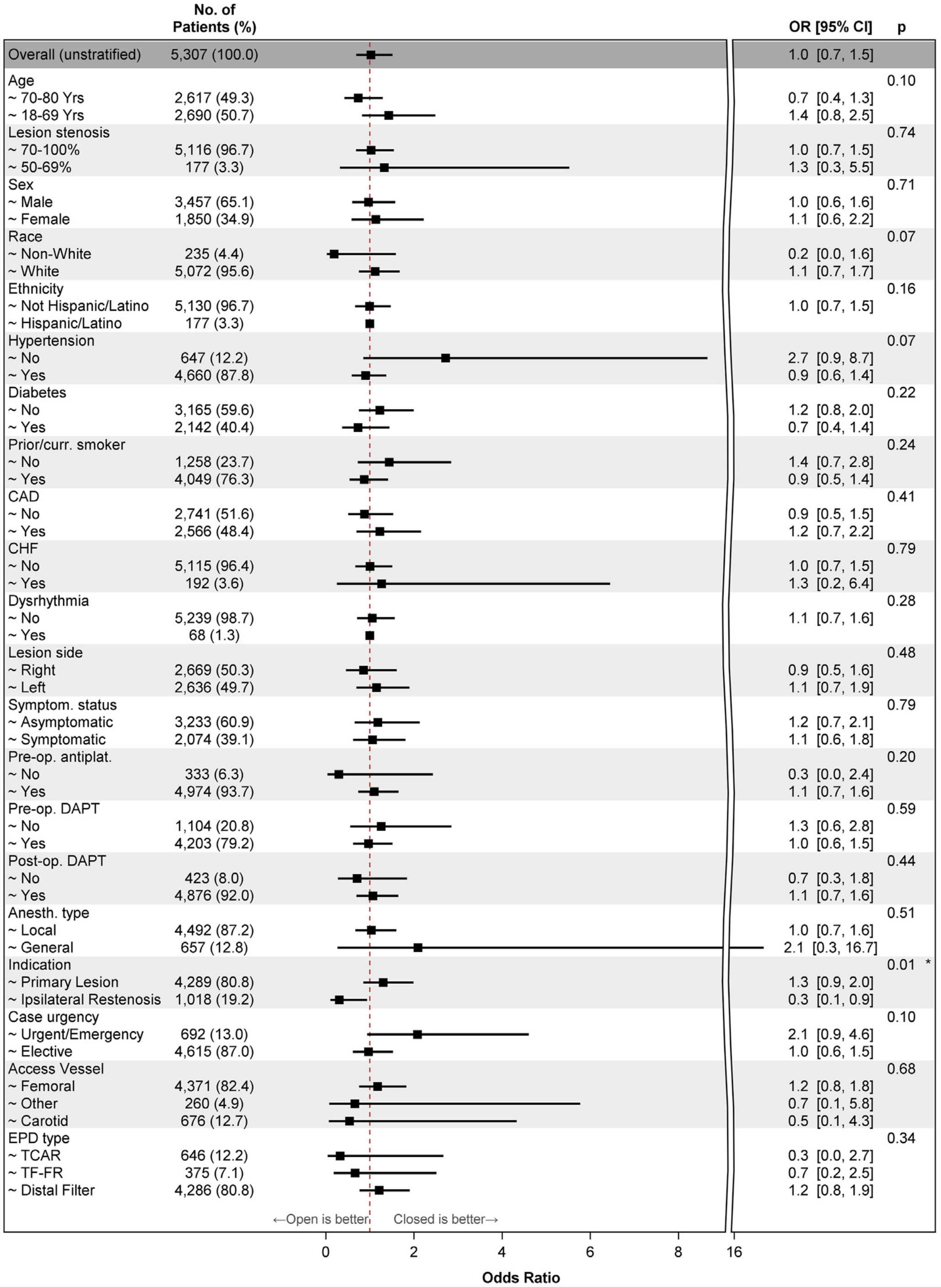

Supplementary Fig (online only). Sub-grouped Forest plot. Forest plot showing the results of subgroup analyses, to examine whether the treatment effect varies across patient subgroups. The top-most row shows the overall (unstratified and unadjusted) treatment effect in the entire analytic cohort. The treatment effect is quantified as an odds ratio ([*OR*] odds of stroke or death [SD] in open-cell group divided by odds in the closed-cell group) shown as a *square marker*. The *horizontal lines* illustrate the 95% confidence intervals (*CIs*). The *dotted vertical line* indicates a null treatment effect (OR, 1, equal odds of SD in both groups). Note that the ORs are plotted on a discontinuous axis, with values 9 to 16 hidden to allow for visualization of extreme values. The *P*-values correspond to Breslow-Day test for homogeneity of ORs, testing the interaction between the treatment (open vs closed stent cell configuration) and each baseline characteristic or procedural detail. Note that only indication has a significant *P*-value, and therefore it was the only effect modifier included in the multivariable regression model. *CAD*, Coronary artery disease; *CHF*, congestive heart failure; *DAPT*, dual antiplatelet therapy; *EPD*, embolic protection device; *TCAR*, transcarotid artery revascularization; *TF-FR*, transfemoral flow reversal.
